# A novel variant of Paganini-Miozzo syndrome: a case report

**DOI:** 10.1093/omcr/omad024

**Published:** 2023-03-25

**Authors:** Roham Sarmadian, Abolfazl Gilani, Habibe Nejad Biglari

**Affiliations:** Infectious Diseases Research Center, Department of Infectious Diseases, Arak University of Medical Sciences, Arak, Iran; Sina Trauma and Surgery Research Center,Department of Surgery, Sina Hospital, Tehran University of Medical Sciences, Tehran, Iran; Neuroscience Research Center, Institute of Neuropharmacology, Department of Pediatric Neurology, Kerman University of Medical Sciences, Kerman, Iran

## Abstract

Paganini-Miozzo syndrome (MRXSPM) is a rare neurogenetic disorder that is transmitted by X-linked recessive inheritance. This is the third case reported of this disease in the world with a novel variant. A boy was referred due to the absence of neck holding and hand tremors. The examinations found facial anomalies. The brain magnetic resonance imaging (MRI) showed cerebral atrophy and diffused white matter, and irregularities were seen in his electroencephalogram (EEG). The echocardiography revealed a mid-muscular ventricular septal defect. A whole exome sequencing (WES) analysis revealed a novel variant (c.979C > T; p.Pro327Ser) of uncertain significance in the HS6ST2 gene indicating Paganini-Miozzo syndrome. The current case provides additional evidence that MRXSPM can be associated with various neurological and cardiac complications. It is essential to rule out other causes, such as metabolic and infectious diseases. EEG, MRI and WES analyses can help to make a definitive diagnosis.

## INTRODUCTION

Paganini-Miozzo syndrome (MRXSPM) is marked by a global developmental deficit, delayed intellectual development, high myopia and minor facial dysmorphism. Paganini-Miozzo syndrome is transmitted by X-linked recessive inheritance. It is caused by a hemizygous mutation on chromosome Xq26 in the heparan sulfate 6-O-sulftransferase2 (HS6ST2) gene [1].

In 2019, Paganini-Miozzo syndrome was detected for the first time in two monozygotic Italian twin boys. Speech and walking were impaired in the two brothers. They had facial, ear, eye and mouth abnormalities [2, 3].

We present the case of a 21-month-old child with MRXSPM-consistent whole exome sequencing (WES). This child is the third case of Paganini-Miozzo syndrome found worldwide with a novel variant.

## CASE PRESENTATION

The child was a 21-month-old boy, first in birth order, born via cesarian section delivery at 38 weeks. The parents were genetically unrelated. His mother did not mention any problem in her pregnancy. The combined test in the 12th week of pregnancy was normal. His birth weight, height and head circumference were 3600 g, 51.5 cm and 35.5 cm, respectively. These variables were currently normal. In the patient’s first-degree family, there was no history of the disease. The patient’s grandmother had six abortions. Also, his cousin had a history of seizures.

**Figure 1:**
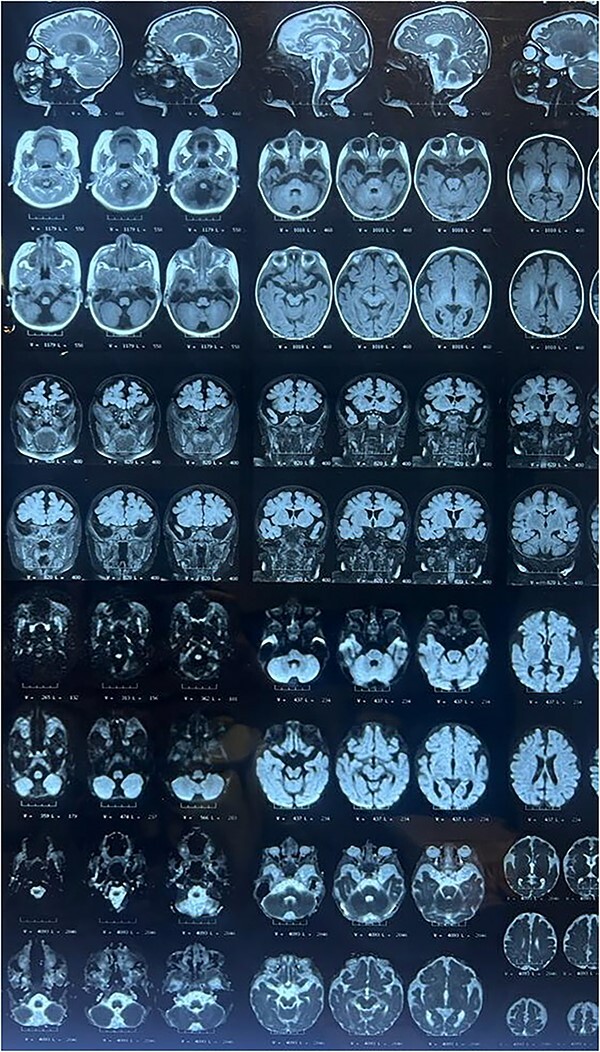
Brain MRI: diffuse white matter in the periventricular, subarachnoid and juxtacortical areas, as well as brain stem, putamen, cerebellum and bilateral thalami involvement.

**Figure 2:**
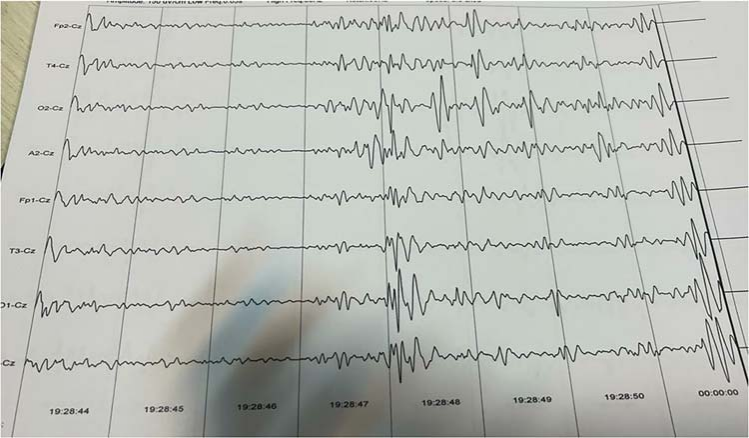
EEG: burst suppression and hypsarrhythmia.

The child’s parents were referred to our clinic at 2 months due to the lack of neck holding and hand tremors. The pediatric neurologist suspected seizures. Brain magnetic resonance imaging (MRI) and an electroencephalogram (EEG) were ordered for the infant. The brain MRI revealed that generalized cerebral atrophy and diffuse white matter in the periventricular, subarachnoid and juxtacortical regions, together with the involvement of the brain stem (posterior to the pons and midbrain), putamen, cerebellum and bilateral thalami. Some of these lesions were diffusion-weighted imaging (DWI)-positive, indicating that a portion of them was acute. Dilated Virchow-Robin spaces were also detected (Fig. 1). Differential diagnoses for the patient included diseases with leukodystrophies such as Krabbe, Mucopolysaccharidoses and other mitochondrial causes. EEG results revealed burst suppression. High-amplitude, irregular and discordant electrical activity, including slow, sharp waves and multi-focal spikes, was also detected, indicating hypsarrhythmia (Fig. 2). Therefore, the patient’s differential diagnosis also included West syndrome. He received Liskantin 250 mg and Levebel 100 mg syrup. After auscultation of extra heart sounds, echocardiography revealed a mid-muscular ventricular septal defect (VSD). The patient had elevated plasma ammonia (113.18 μmol/L) and alanine and aspartate aminotransferases (53 and 48 U/L, respectively). Metabolic tests were normal. Due to the elevated levels of cytomegalovirus (CMV) immunoglobulin M (IgM) in the tests (11.25 g/L, normal < 9), a pediatric infectious diseases specialist performed a lumbar puncture with the suspicion of central nervous system infection by CMV, which revealed a normal cerebrospinal fluid analysis and the absence of an infection. In addition, both the eye exam for chorioretinitis and the auditory brainstem response test were normal. Finally, a WES analysis was performed for the patient that revealed a novel variant (c.979C > T; p.Pro327Ser) of uncertain significance in heparan sulfate 6-O-sulftransferase2 (HS6ST2) gene. Therefore, the patient was diagnosed with Paganini-Miozzo syndrome. Despite the aforementioned medications, convulsions continued and progressed to numerous times a day. Clobazam 10 mg half-tablet every 8 hours was added. In addition, subcutaneous or intramuscular adrenocorticotropic hormone (ACTH) ampoule 1 U/kg (via 100 unit insulin syringe) was prescribed daily for 14 days, then every other day for the next 14 days and once every 3 days for the third 14 days to taper off. Currently, the child’s seizures are well controlled.

**Figure 3:**
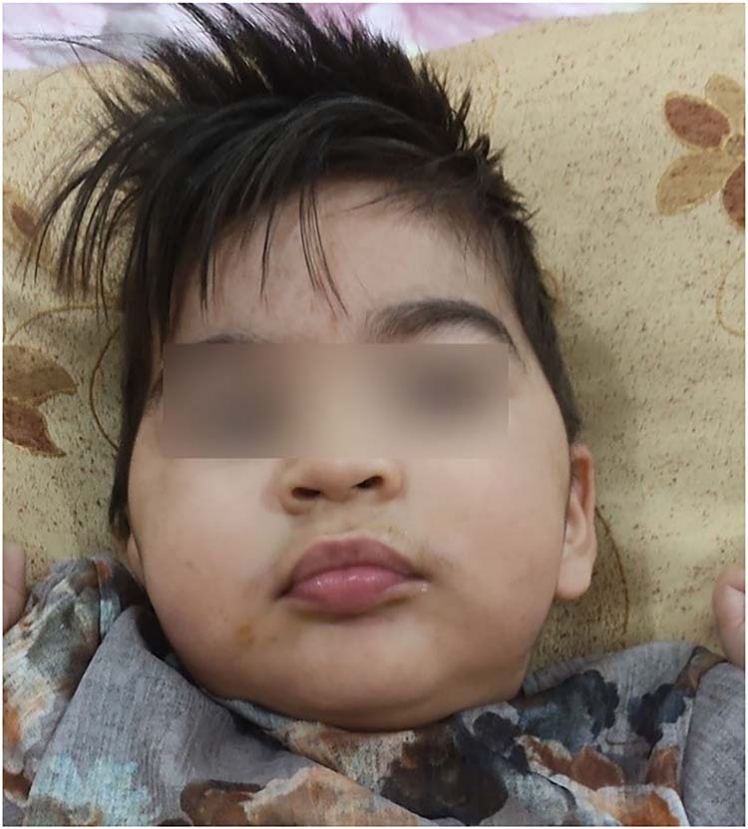
Patient’s appearance: hypertelorism, depressed nasal bridge and arched eyebrows.

In the examination, hypertelorism, depressed nasal bridge and arched eyebrows were observed (Fig. 3). Currently, the child can hold his neck, but he cannot sit or walk and can only crawl with outstretched hands. He can make sounds, but cannot articulate simple words.

The child undergoes echocardiography every 6 months, electrocardiography every 3 months, as well as a pediatric neurologist visit every 3 months.

## DISCUSSION

MRXSPM was first discovered in Italy, by Paganini *et al*. [2] in two monozygotic twin brothers. The present report is the second case report of MRXSPM.

The two Italian cases were referred for intellectual disability. Our patient similarly had delayed developmental milestones such as neck holding, intellectual and walking impairment. Our patient’s seizures manifested themselves as hand tremors. At 1 year old, the Italian boys had febrile convulsions that resolved. Our patient had frequent seizures even after 1 year of age, which were controlled by medication. Italian twins had facial, gastrointestinal and genitourinary issues. Except for facial anomalies, our patient’s physical exams were normal.

The two brothers were delivered at 30 weeks due to fetal distress and were born from a triplet dichorionic-tri amniotic pregnancy resulting from *in vitro* fertilization. In contrast, our case involves a singleton born of a natural, full-term pregnancy.

The Italian twins’ epileptiform EEG showed diffuse and irregular spikes. Our patient’s EEG showed hypsarrhythmia and burst suppression. Italian brothers’ brain MRIs showed mild lateral ventriculomegaly. Our MRI showed modest lateral ventricular enlargement, white matter diffusion and extensive atrophy. The Italian brothers’ lab tests showed high serum lactate, elevated alanine and low glycemic scores. In our patient’s laboratory tests, these values were normal. In addition, plasma ammonia levels were increased in our patient. The WES analysis of Italian twins detected the mutation c.916G > C in the X-linked HS6ST2 gene. However, our patient had a novel variant (c.979C > T).

Given the small number of Paganini-Miozzo syndrome cases worldwide, it is currently not possible to form a definitive assessment of the condition’s defining features.

## CONCLUSION

In MRXSPM, there is evidence of developmental milestone delays and minor anomalies. In addition, these patients may experience seizures, which manifest as irregular spikes on EEG. The MRI of these patients reveals brain atrophy, ventricular enlargement or diffuse white matter.

In general, children referred with neurodevelopmental issues and seizures, as well as similar MRI or EEG abnormalities, should be examined initially for metabolic and infectious diseases. If the aforementioned diagnoses were ruled out, WES should be performed to examine genetic disorders and epileptic syndromes. The mutation in the X-chromosome HS6st2 gene indicates MRXSPM.

## Supplementary Material

Sup1_omad024

Sup2-word_omad024

CARE-checklist-English-2013_omad024

## Data Availability

Not applicable.
