# Supplementary figures and images for: A novel variant of Paganini-Miozzo syndrome: a case report
Source: Oxf Med Case Reports. 2023 Mar 25;2023(3):omad024. doi: 10.1093/omcr/omad024 (PMC10041959; doi:10.1093/omcr/omad024)

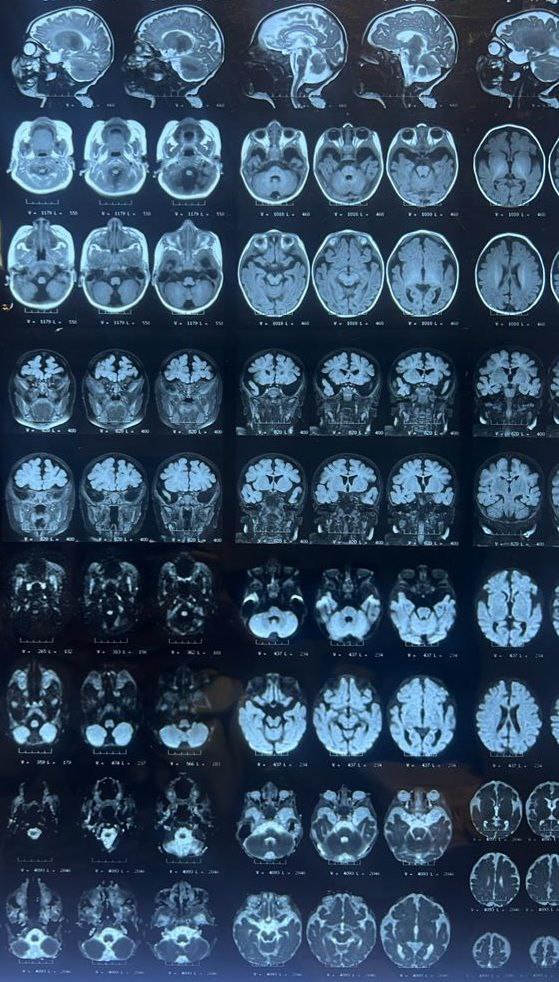


1


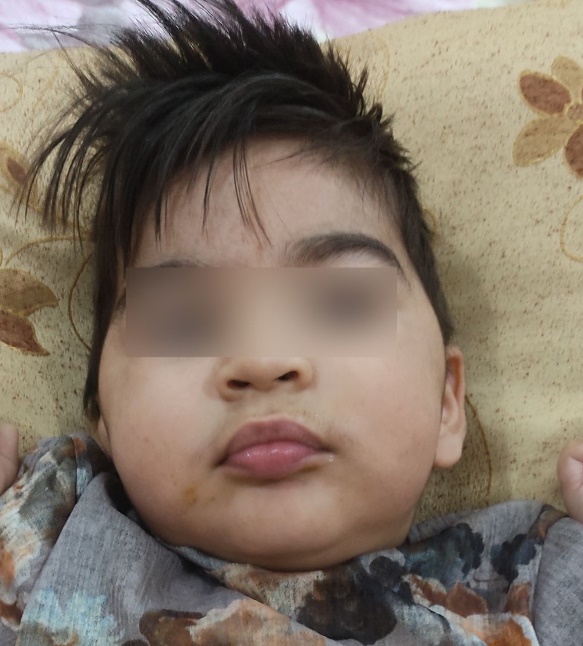


2

Supplement: Sup1_omad024 [file sup1_omad024.docx]

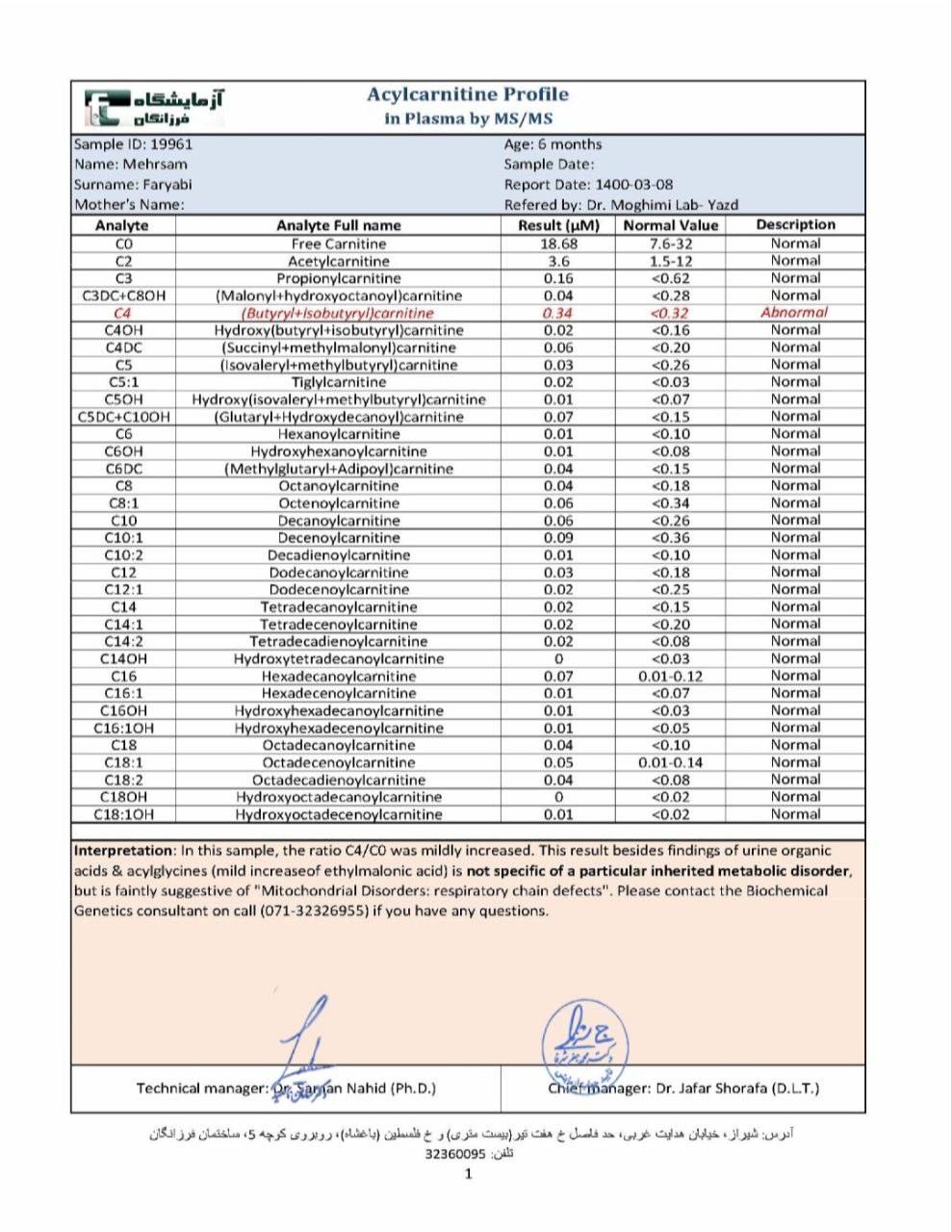


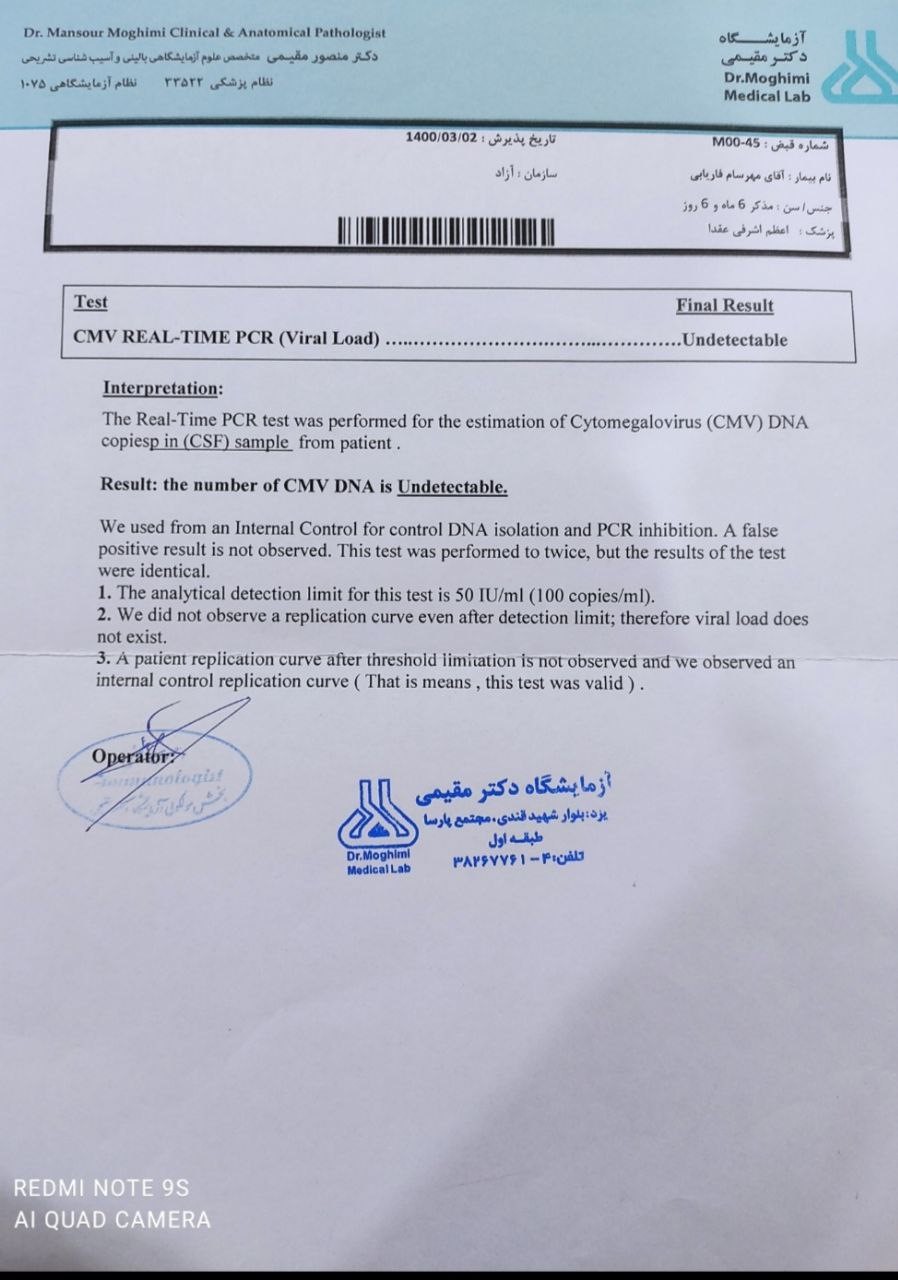


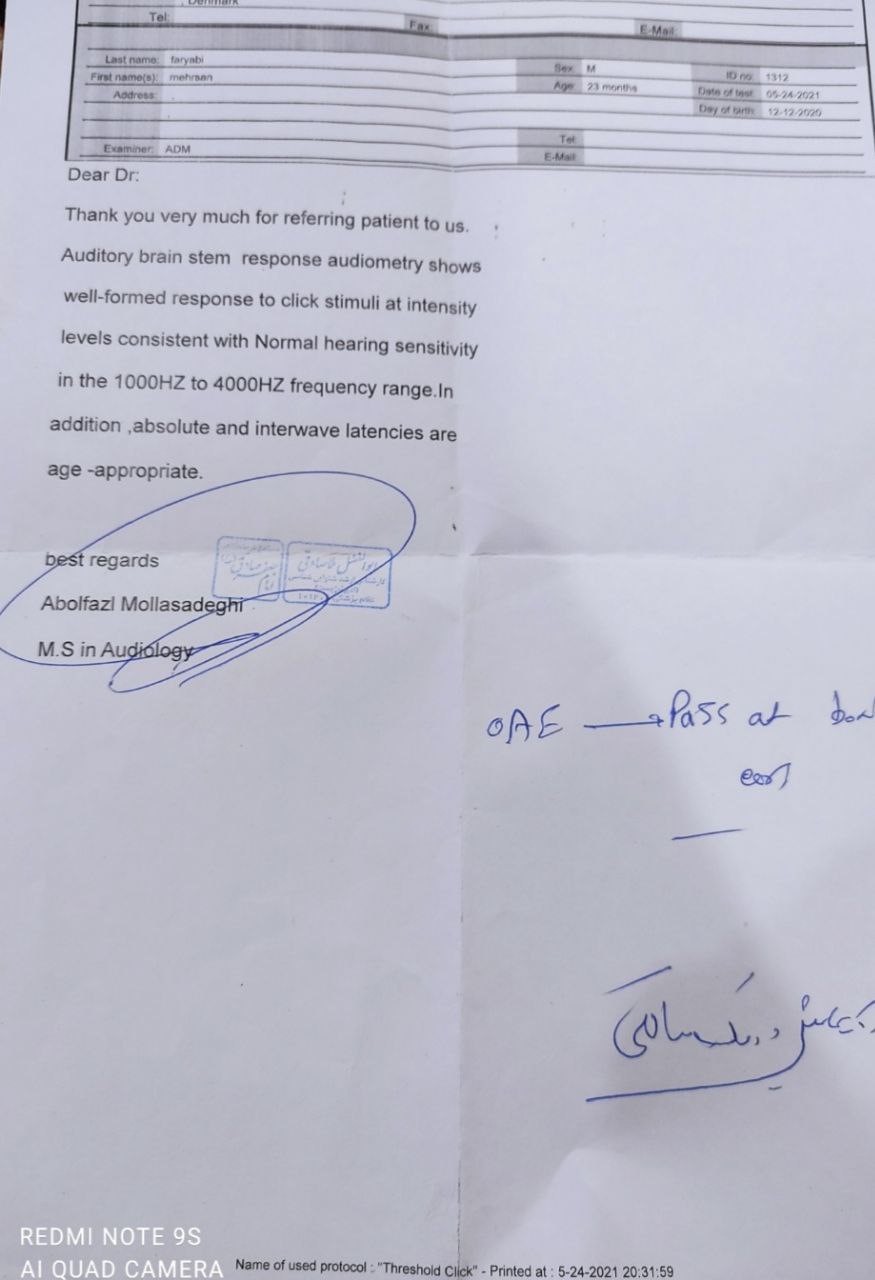


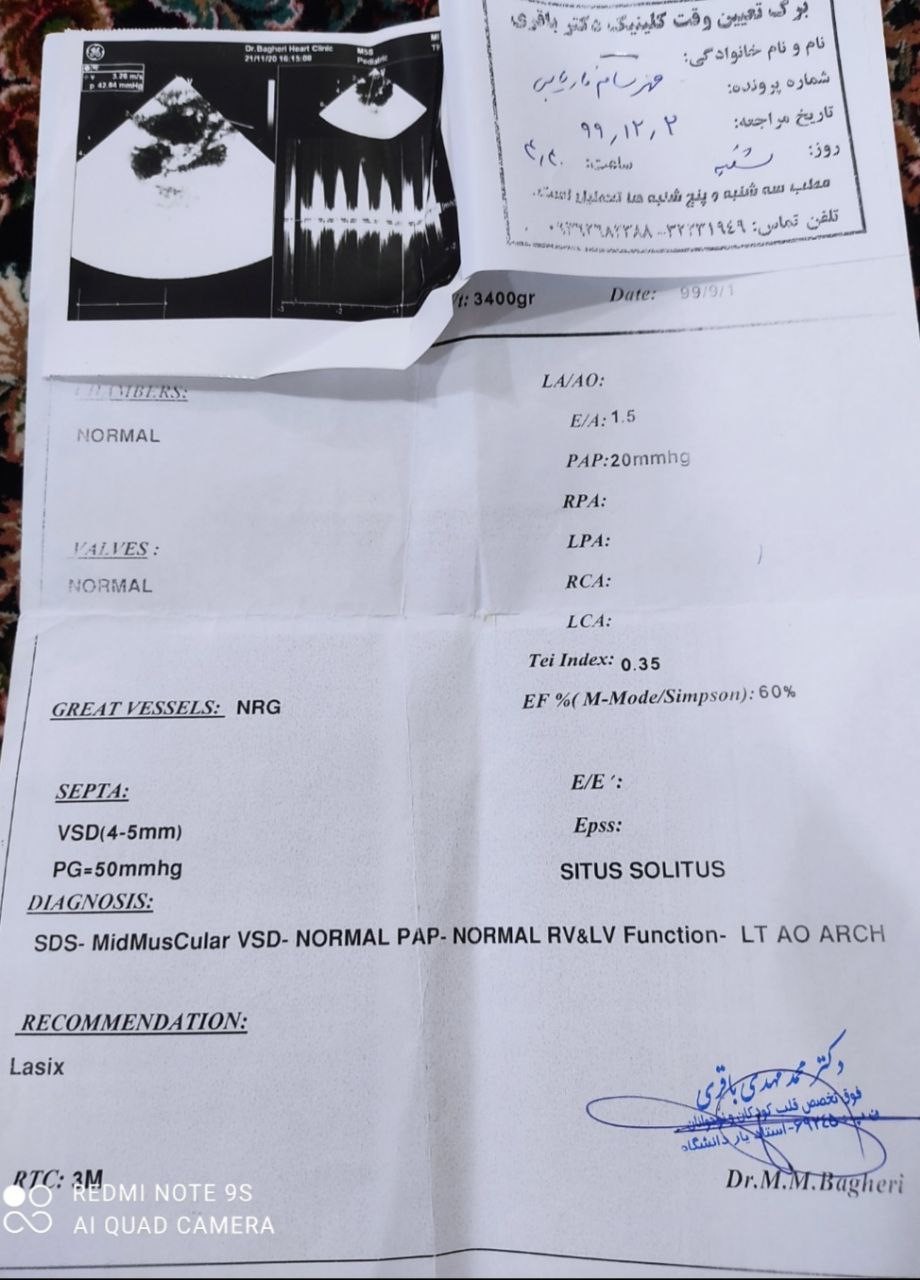


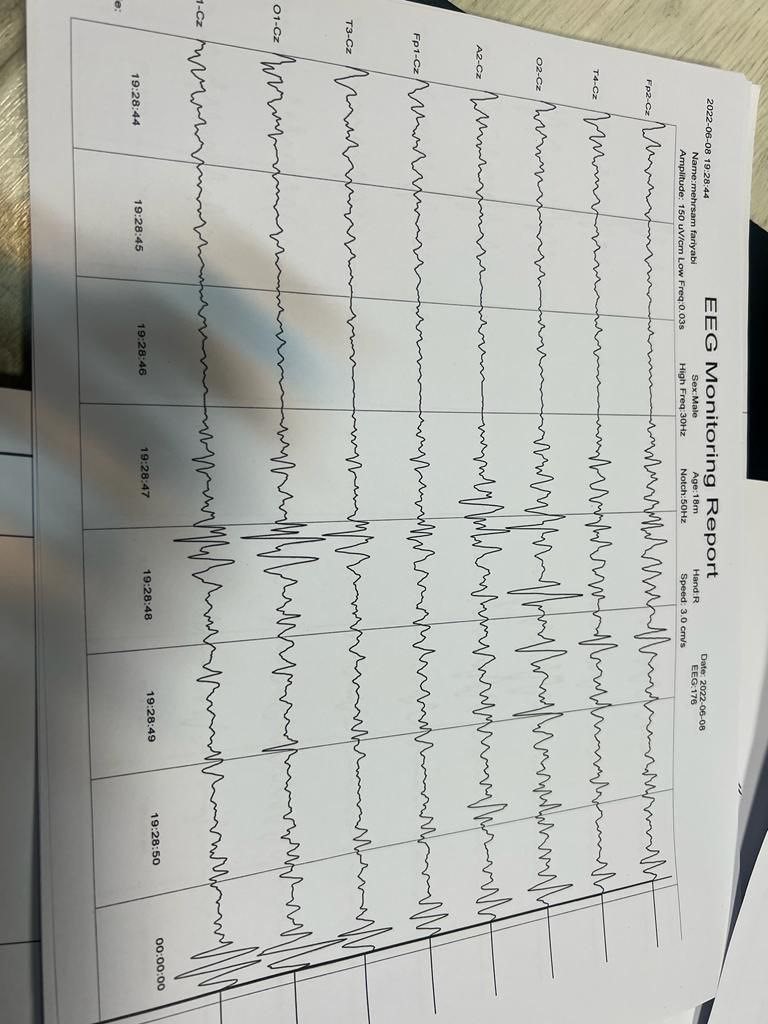


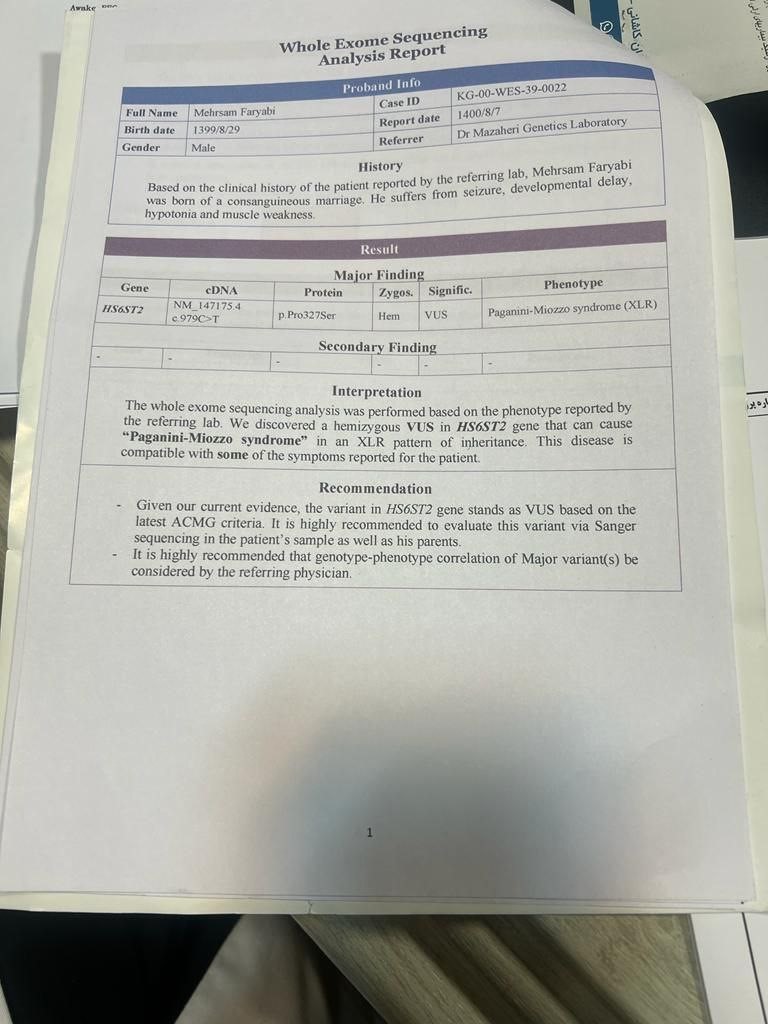


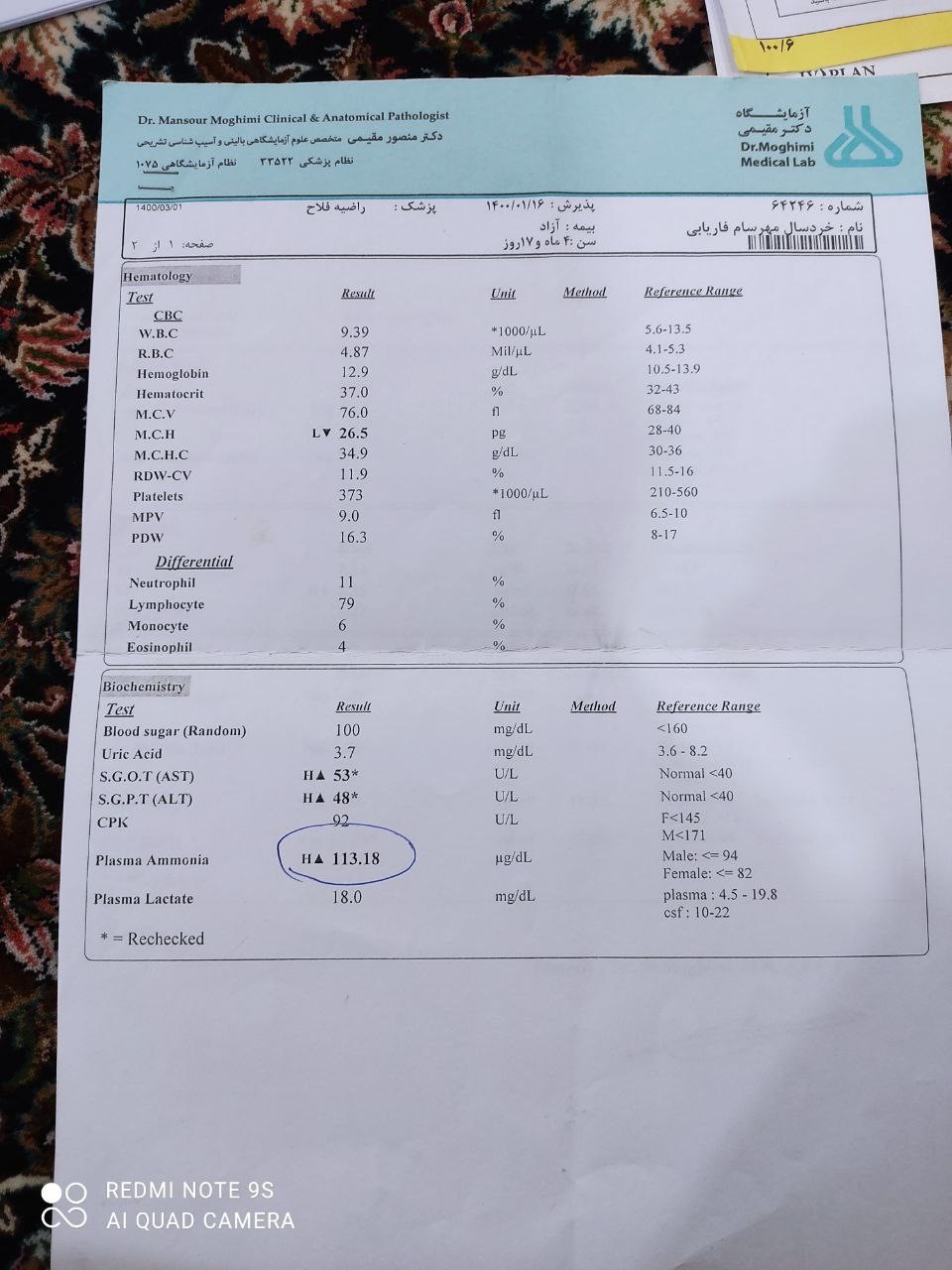


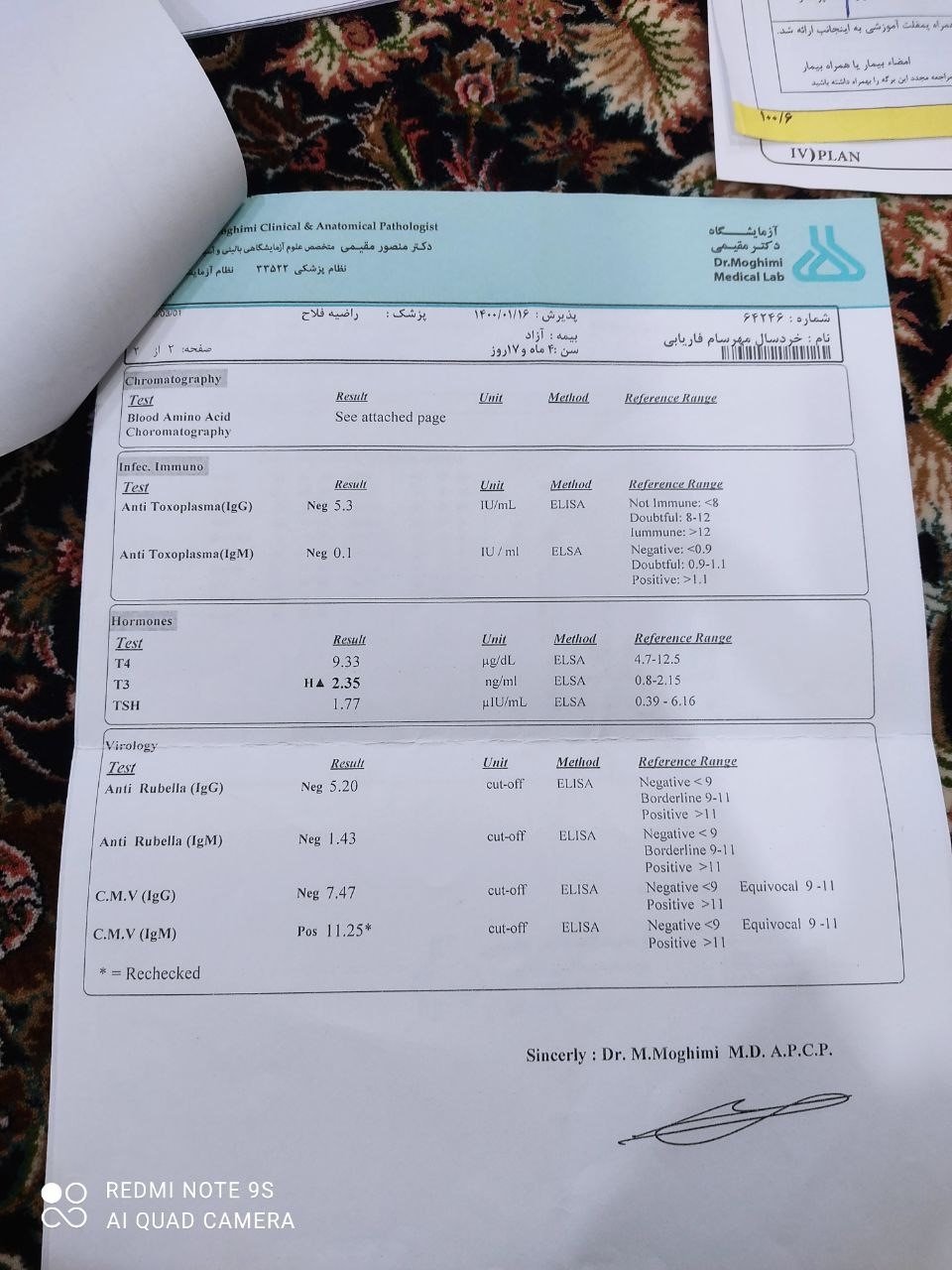


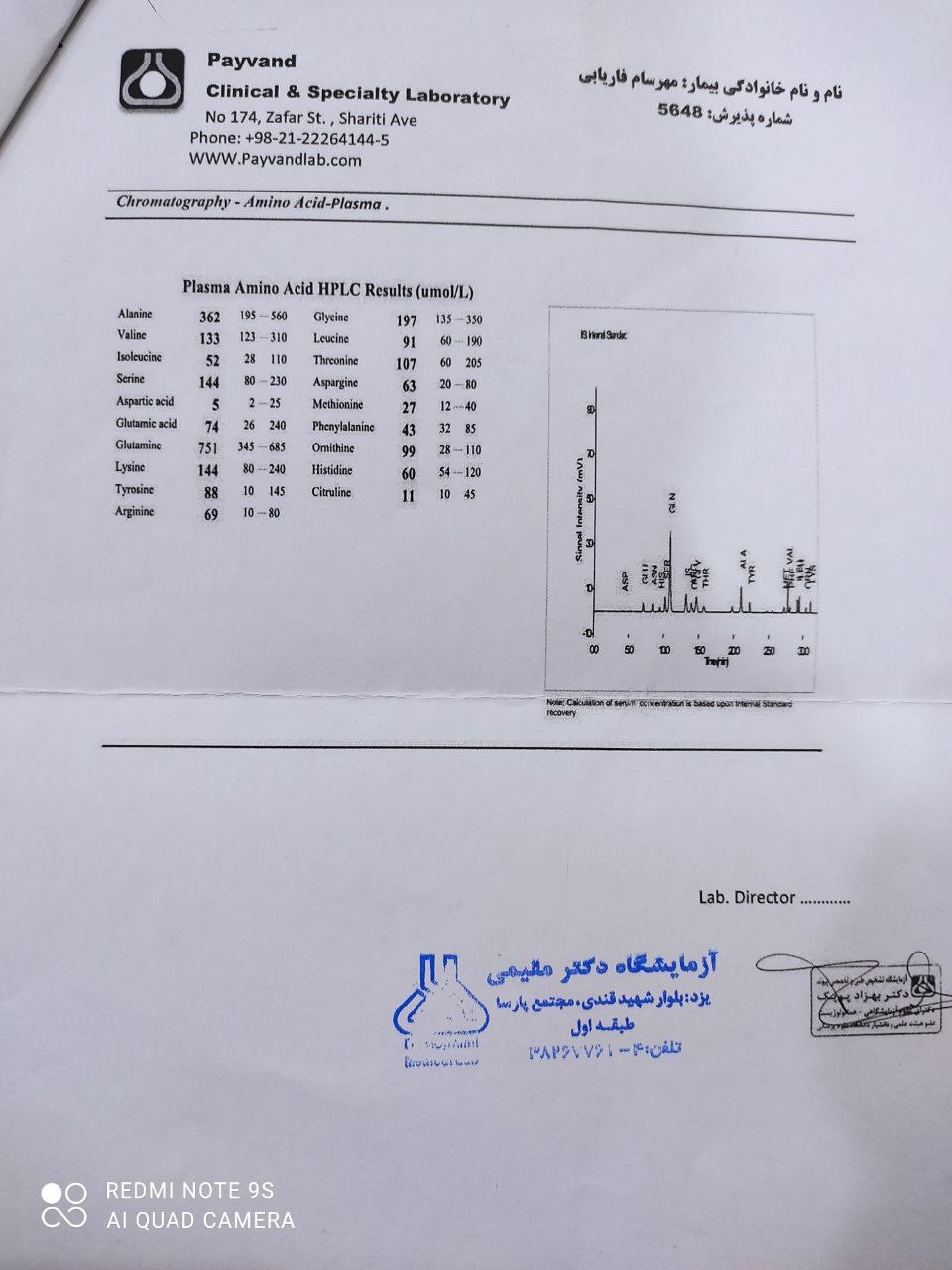

Supplement: Sup2-word_omad024 [file sup2-word_omad024.docx]
